# Exosome Biomarker Profiling Using a Paper-Based Vertical Flow Assay

**DOI:** 10.3390/bios15100694

**Published:** 2025-10-14

**Authors:** Arnau Pallarès-Rusiñol, Jennifer Marfà, Rosanna Rossi, Mercè Martí, María Isabel Pividori

**Affiliations:** 1 Grup de Sensors i Biosensors, Departament de Química, Universitat Autònoma de Barcelona, 08193 Bellaterra, Spain; arnau.pallares@uab.cat (A.P.-R.); jennifer.marfa@autonoma.cat (J.M.); rosanna.rossi@uab.cat (R.R.); 2 Biosensing and Bioanalysis Group, Institute of Biotechnology and Biomedicine, Universitat Autònoma de Barcelona, Bellaterra 08193, Spain; merce.marti@uab.cat

**Keywords:** exosome profiling, vertical flow assay, breast cancer, alkaline phosphatase, liquid biopsy, paper-based immunoassay, flow cytometry

## Abstract

Exosomes are nanoscale extracellular vesicles that carry valuable biomolecular information. However, their characterization still depends on complex and costly techniques such as flow cytometry. In this study, a paper-based Vertical Flow Assay (VFA) specifically designed for the detection and profiling of exosomes derived from metastatic breast cancer cell lines is presented. The assay operates in an ELISA-like format, targeting exosomal surface proteins (CD9, CD63, CD81, and EGFR1) with specific antibodies and a secondary antibody conjugated to alkaline phosphatase. Upon reaction with the NBT/BCIP substrate, an insoluble indigo precipitate forms on the nitrocellulose membrane, generating a visual signal that can be further quantified by smartphone imaging. The VFA was optimized for membrane type, pore size, and blocking agents, reaching a detection limit of ~6 × 10^7^ exosomes µL^−1^ in less than 20 min. Comparative studies with bead-based flow cytometry confirmed consistent biomarker expression profiles, demonstrating the reliability of the method. By enabling exosome biomarker profiling in a simplified and low-cost format, this approach provides a promising alternative to flow cytometry and other applications required for exosome characterization.

## 1. Introduction

The characterization of exosomes and other extracellular vesicles (EVs) is a challenging task due to their nanometric sizes that makes them out of the sensitivity range of most cell-oriented analysis platforms. Most of the techniques require extensive pre-treatment protocols, as well as the use of expensive equipment and skilled personnel. Despite this, portable devices have been reported, mainly based on electrochemical readouts [1,2,3]. Still, there is a lack of RDTs (Rapid Diagnostic Tests) following the REASSURED criteria [4] for the detection of EVs. Accordingly, the development of paper-based platforms for exosomes could provide interesting analytical features in a cost-effective approach. Lateral Flow Assay (LFA) and Vertical Flow Assay (VFA) are based on the reaction of the analyte with a signal-generating system, which provides a naked-eye readout integrated on a cellulose membrane and in a simple-to-use approach [5]. The use of nanomaterials as gold nanoparticles or fluorescent beads is usually implemented for enhancing visual signals and improving the LODs [5,6]. In addition, the use of portable readers to obtain quantitative information enhances analytical capabilities, and, in some instances, similar LODs than biosensing devices can achieve [7]. There are commercially available LFA readers, such as iPeak (IUL Instruments, Barcelona, Spain) and ESEQuant Flex (Dialunox, Stokach, Germany), just to mention a few. Even more interesting, nowadays, the camera of almost any smartphone can be used for optical readout. Many examples have been published, mostly with LFA, but also with VFA in different applications and in a variety of signal-generating systems [8,9,10,11].

In the case of exosomes, the detection by LFA presents some intrinsic issues related to active exosome movement through the hydrophilic membrane because of their bilipidic membrane [12]. Moreover, the heterogeneity of EV in the samples can be a handicap for the uniform mobility of the front line. Still, some attempts were reported. Mainly, they are based on LFA targeting the membrane proteins of exosomes, usually ubiquitous markers such as tetraspanin receptors CD9, CD63, and CD81 [13,14,15,16,17,18] and showing LODs of around 10^7^ exosomes µL^−1^. As a shortcoming of those approaches, most of the devices are not fully integrated LFA strips. In order to prevent non-specific adsorption, those approaches are based on non-traditional LFA formats in which the signal-generating system is not stored in an integrated pad but preincubated with the exosomes aside from the strip, in a tube or microplate, in which the strip is then dipped in.

In this study, the challenges associated with the mobility of EVs on paper-based platforms are addressed by using hydrophobic interactions between EVs and nitrocellulose membranes. A novel approach based on a VFA, a paper-based platform inspired by the classic dot blot immunoassay [19], is presented. Unlike LFA, where liquid flows parallel to the membrane, VFA operates with the flow of liquid perpendicular to the membrane layers, significantly increasing the speed of the assay [20]. This enhanced flow, driven by both capillarity and gravity, allows for a more rapid analysis compared to LFA, reducing assay times significantly. While traditional VFAs often rely on biologically modified gold nanoparticles for detection [20,21,22,23], this work explores an enzymatic signal-generating system using alkaline phosphatase (ALP) as a label [24], which presents a competitive advantage in terms of cost-effectiveness and simplicity over nanoparticle-based systems. This study details the design, construction, and optimization of the VFA for the detection of exosomes derived from breast cancer cell lines, specifically using ALP for signal generation.

An ELISA-like VFA format is proposed as a semi-quantitative test for the determination of membrane proteins in exosomes, using a smartphone for the reading of the visual signals. In addition to quantification, the profiling of exosome surface biomarkers—such as membrane proteins CD9, CD63, CD81, and EGFR1—is of critical importance for cancer research. Profiling the biomarkers present on the surface of exosomes provides crucial insights into cell–cell communication, tumor progression, and metastasis, making it an important tool for early detection [25]. The VFA is also explored to perform this biomarker profiling of exosomes. By combining rapid detection and profiling capabilities, this method offers a cost-effective, portable alternative to more traditional techniques like flow cytometry, which often requires more complex instrumentation and expertise [25].

This study demonstrates that the proposed ELISA-like Vertical Flow Assay enables reliable detection and the profiling of breast cancer-derived exosomes, showing good agreement with flow cytometry while providing a simpler, faster, and cost-effective alternative for biomarker characterization.

## 2. Materials and Methods

### 2.1. Instrumentation

**Figure 1:**
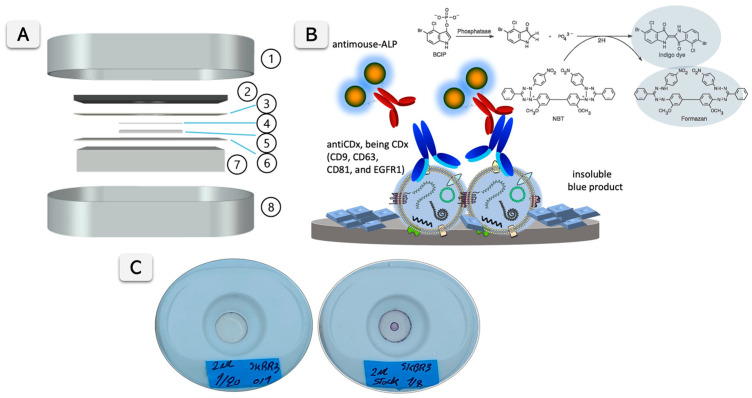
(**A**) presents a schematic representation of the Vertical Flow Assay (VFA) cartridge components, featuring the cover (1) and support (8) of the MedMira cassette. The plastic support of the commercial cassette is shown in (2). Various membranes are affixed to the plastic support with double-sided tape (3), including a nitrocellulose membrane (4), a medium-weight cotton linter pad (5), and a filter paper layer (6). An absorption cotton linter thick pad (7) is positioned beneath the plastic support. (**B**) illustrates the detection mechanism of membrane proteins on exosomes in an ELISA-like format, based on anti-CDx antibodies (CD9, CD63, CD81, and EGFR1) and a secondary antibody conjugated with alkaline phosphatase (ALP). The ALP catalyzes a reaction leading to the formation of an insoluble blue product, visualized as a positive signal. (**C**) shows an image of a VFA cartridge displaying color development of a positive test after 15 min of reaction time, indicating the presence of biomarkers.

Nanoparticle tracking analysis (NTA) was performed using the NanoSight LM10-HS system with a tuned 405 nm laser (NanoSight Ltd., Malvern, UK). Spectrophotometric measurements were performed on a Tecan Infinite m200 PRO (Tecan Group Ltd., Männeford, Switzerland) microplate reader controlled by Magellan v7.0 software. Flow cytometry was performed using Cytoflex LX (Beckman Coulter Inc, Indianapolis, IN, USA) and analyzed with integrated software Cytexpert v.2.4 and FlowJo analysis software (FlowJo LLC, BD, Franklin Lakes, NJ, USA). In addition, for the visual signal quantification, pictures from the VFA cartridges were taken with a 12 megapixels smartphone camera at 23 cm, using an LED illumination support with 1100 Lm intensity and a color temperature of 6000–6500 K. The pictures were treated and analyzed using the gel analysis tool from ImageJ Fiji v.1.53e [26]. The cartridges for the Vertical Flow Assay devices (RVF, VF-1-01) were purchased from MedMira (Halifax, MA, USA). Different membranes were tested in the VFA cartridges. Figure 1A shows the different components of the VFA cartridge. All details of VFA construction and materials are provided in Supplementary Information (SI) Section S1 (Figure S1 and Table S1).

### 2.2. Chemicals and Biochemicals

Calf intestine alkaline phosphatase enzyme (ALP, cat. no. 10713023001) was purchased from Roche Diagnostics (Merck kGaA, Darmstadt, Germany). The substrates for ALP used were NBT/BCIP (1-Step NBT/BCIP Substrate Solution, cat. no. 34042) from Thermo Fisher Scientific (Waltham, MA, USA). Magnetic particles (MPs) tosylactivated (Dynabeads™ M450 Tosylactivated, cat. no. 14013) were purchased from Thermo Fisher Scientific (Waltham, MA, USA). The mouse monoclonal antibodies against tetraspanins, antiCD9 (cat. no. 10626D), antiCD63 (cat. no. 10628D), antiCD81 (cat. no. 10630D), and goat polyclonal antiMouse ALP conjugate (cat. no. 31320) was purchased from Thermo Fisher Scientific. The mouse monoclonal antibody against specific EGFR1 (epithelial growth factor receptor, type 1) protein (cat. no. ab30) and Cy5-labeled goat anti-mouse (anti-mouse-Cy5, cat. no. ab97037) were purchased from Abcam (Cambridge, UK). For the cell culture, Dulbecco’s Modified Eagle’s Glutamax (DMEM, cat. no. 31966-021) medium and fetal bovine serum (FBS, cat. no. 26140079) were purchased from Gibco (Thermo Fisher Scientific). For the protein quantification, Pierce BCA Protein Assay kit (cat. no. 23227) was purchased from Thermo Fisher Scientific. All other reagents were in analytical reagent grade. The composition of the solutions is described in Section S1 (SI).

### 2.3. Cell Culturing, Exosome Isolation, and Purification

Breast cancer cell lines SKBR3 (ATCC HTB-30, American Type Culture Collection, Manassas, VA, USA) and MDA-MB-231 (ATCC HTB-26, American Type Culture Collection, Manassas, VA, USA) were grown as described in Section S2 (SI). Exosomes were purified from cell culture supernatant by differential ultracentrifugation, as previously reported by our research group with minor changes [2]. Detailed information on the differential ultracentrifugation procedure, including centrifugation speeds, *g*-forces, and durations of each step, is outlined in Figure S2 (Section S2). Exosomes were resuspended in 10 mmol L^−1^ tris buffer solution (pH 7.4, 0.22 µm sterile filtered) and stored at –21 °C.

### 2.4. Characterization of Exosomes by Nanoparticle Tracking Analysis, Cryogenic Transmission Electron Microscopy, and BCA Protein Assay

The size distribution and concentration of particles were estimated by nanoparticle tracking analysis (NTA), as described in Section S3 (SI). The purified exosomes were diluted in filtered PBS buffer solution between 500 and 10,000-fold, depending on the sample’s initial concentration [2]. Nanosight NTA software analyzed raw data videos by triplicate during 60 s with 25 frames s^−1^. Cryogenic TEM images were collected by a Jeol JEM 2011 (JEOL USA Inc., Peabody, MA, USA) transmission electron microscope at an accelerating voltage of 200 kV [2,27]. Exosome suspensions were vitrified using standard procedures: samples were deposited on glow-discharged carbon grids, blotted to form a thin film, and plunge-frozen in liquid ethane with a Vitrobot system. Exosomes were maintained at −182 °C with liquid ethane during the whole process. Grids were imaged under liquid-nitrogen conditions at low electron dose to preserve vesicle morphology. The total protein concentration of the exosomes was estimated using the Bicinchoninic acid protein assay (BCA), following the manufacturer instructions, using bovine serum albumin (BSA) standards in tris buffer solution. The spectrophotometric measurement was performed at 562 nm using a Tecan Infinite m200 PRO microplate reader [2].

### 2.5. Exosome Biomarker Profiling by Bead-Based Flow Cytometry

Flow cytometry was used to characterize the presence of membrane protein markers of interest on the surface of exosomes derived from SKBR3 and MDA-MB-231 breast cancer cell lines. Specifically, the presence of tetraspanin receptors CD9, CD63, and CD81, and epithelial-specific EGFR1 receptor [28] was evaluated. The flow cytometry assay is based on the immobilization of exosomes on the surface of magnetic particles to increase its size within the resolution of the flow cytometer [29]. To achieve that, 3.5 × 10^10^ exosomes were covalently immobilized on 1.6 × 10^7^ MPs, as described in detail in Section S4 (SI), followed by indirect labeling with antiCDX (mouse) and anti-mouse Cy5 secondary antibody (being CDX either CD9, CD63, CD81, or EGFR1 biomarkers).

### 2.6. Optimization of Vertical Flow Assay Design and Experimental Parameters

The construction of VFA was carefully optimized, including all membranes and materials, to improve the flow rate, as schematically shown in Figure 1A, to enhance the sensitivity of the assay and to reduce non-specific background signals. As model analytes for optimization experiments, ALP enzymes and exosomes derived from SKBR3 cell line were used. Different nitrocellulose membranes were tested, including unbacked nitrocellulose membranes for LFA of increasing capillarity flow rates (AE98, AE99, and AE100) and cellulose nitrate membranes (NC membranes) of 0.1, 0.2, and 0.45 µm pore sizes. In addition, Protran BA85 (0.45 µm pore size) membrane was also tested as standard blotting nitrocellulose membrane. Figure S1 and Table S1 (SI)  provide all details from the materials tested. Different protein and non-protein agents were tested for blocking, as described in Section S1 (SI). As model analytes, different dilutions of ALP (500 mU mL^−1^, 166 mU mL^−1^, and 55 mU mL^−1^) were tested. In addition, other experimental parameters, such as immobilization time, the cellulose membrane used as sample pad, and the addition of a non-absorbent separation layer, were studied. The optimization experiments were performed following the standard protocol detailed in Section S5 (SI) for testing of all nitrocellulose membranes (Table S1, SI) and blocking solutions.

### 2.7. Vertical Flow Assay for Exosome Biomarker Quantification and Profiling

The determination of the surface proteins of the EVs was performed on a VFA nitrocellulose membrane in an ELISA-like format with indirect labeling, as shown in Figure 1A,B, and described in detailed in Section S6 (SI).

Briefly, in this assay, mouse monoclonal antibodies against tetraspanins CD9, CD63, and CD81, and epithelial-specific EGFR1 were used to react with the immobilized exosomes. As the secondary antibody, an anti-mouse–ALP conjugate antibody was used. Finally, the readout was achieved by adding 10 µL of NBT/BCIP substrate. The substrate combines nitro blue tetrazolium chloride (NBT) and 5-bromo-4-chloro-3′-indolyphosphate p-toluidine (BCIP). After just 15 min of reaction, the membranes were washed. The phosphate of BCIP is hydrolyzed by ALP, producing an intermediate that dimerizes to indigo dye upon oxidation with NBT, which is reduced to NBT-formazan. Both reaction products create an intense blue–purple precipitate on the surface of the VFA, which can be easily visualized with the naked eye (Figure 1C). Besides the visual detection, the analytical performance of the assay was determined by imaging the colored dots using a smartphone camera under white LED light. The readout can be achieved with the naked-eye or, instead, imaging with a smartphone camera for further processing, as outlined in Section S7 (SI). In any case, images were acquired using a standard, commercially available smartphone camera equipped with a 12-megapixel rear sensor. The working range of this assay was estimated by creating a calibration plot with exosomes derived from SKBR3 and MDA-MB-231, detected by antiCD81 as the universal exosome marker, and anti-mouse–ALP conjugate.

The range of concentrations comprises 0 to 3 × 10^8^ particles µL^−1^, according to NTA count. To assess the specificity of the assay, different antibodies were tested with exosomes derived from SKBR3 and MDA-MB-231. Mouse monoclonal antibodies specifically against tetraspanins (CD9, CD63, and CD81) and epithelial-specific biomarker EGFR1 were used. Besides the negative controls, other controls were tested to determine the signal of the intrinsic ALP activity from the exosomes [1], performed as the positive test but avoiding adding of the primary and secondary antibody.

The statistical analyses were performed using GraphPad v10.6.0 (Boston, MA, USA). The value *p* < 0.05 was considered significant.

### 2.8. Safety Considerations

All works were performed in a Biosafety cabinet, and all material was decontaminated by autoclaving or disinfected before discarding following U.S. Department of Health and Human Services guidelines for level 2 laboratory Biosafety [30].

## 3. Results

### 3.1. Characterization of Exosomes by Nanoparticle Tracking Analysis, Cryogenic Transmission Electron Microscopy, and BCA Protein Assay

**Figure 2:**
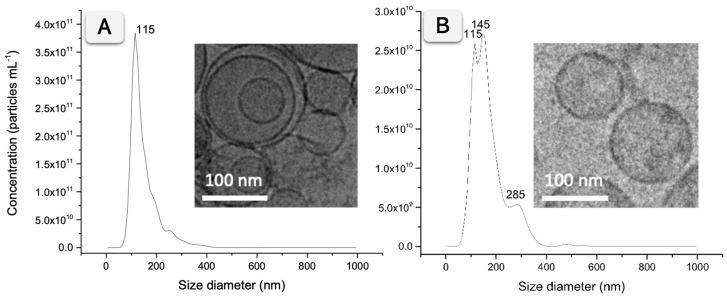
Characterization by NTA and Cryo-TEM micrographs of purified EV samples from SKBR3 (**A**), MDA-MB-231 (**B**) breast cancer cell lines. The NTA characterization analyzed raw data videos by triplicate during 60 s with 25 frames per second and the temperature of the laser unit set at 24.8 °C. Cryo-TEM images were obtained at an acceleration voltage of 200 kV.

The NTA of exosomes derived from SKBR3 and MDA-MB-231 cell lines revealed a similar size distribution. Figure 2A summarizes the results, with a size distribution histogram showing a peak at 115 nm for SKBR3 exosomes. In the case of MDA-MB-231 exosomes, two peaks at 115 and 145 nm and a smaller peak at 285 nm corresponding to small aggregates are shown, as depicted in Figure 2B. The exosomes from SKBR3 and MDA-MB-231 were further imaged by Cryo-TEM, confirming the presence of individual vesicles and small aggregates ranging from 50 to 400 nm. The insets in Figure 2 show representative vesicles corresponding to the predominant population observed in the NTA histogram, while a wider Cryo-TEM field, including a few small aggregates, indicated by arrows, is presented in Figure S3 (SI).

Besides the size distribution, the particle and protein concentration were estimated by NTA and BCA protein assay, respectively. Exosomes derived from SKBR3 showed a higher concentration (2.69/SD 0.19 × 10^12^ particles mL^−1^ and 1.038 mg mL^−1^ total protein) than MDA-MB-231 (3.24/SD 0.04 × 10^11^ particles mL^−1^ and 0.324 mg mL^−1^ total protein). Table S2 (SI) summarizes the results.

### 3.2. Exosome Biomarker Profiling by Bead-Based Flow Cytometry

The flow cytometry assay was performed by staining the exosomes immobilized on magnetic particles in a bead-based cytometry format, as previously described by our research group [1,2,3,29]. Figure S4 (SI) shows the results of the bead-based flow cytometry analysis on exosome surface biomarkers. Figure S4A (SI) shows the dot blots, while Figure S4B shows the histograms obtained from the bead-based flow cytometry assay of exosomes derived from SKBR3 and MDA-MB-231. As expected, tetraspanins CD9, CD63, and CD81, considered as the general biomarkers of exosomes, show strong signals in both cell lines, with varying intensities for CD63 and EGFR1. Negative controls are included: (-) Control A (without exosomes) and (-) Control B (without a primary antibody), both of which show minimal or no signal detection. Regarding the specific epithelial marker EGFR1, its presence was also confirmed in both samples, with 13% in SKBR3 exosomes and 37% in MDA-MB-231 exosomes being EGFR1 positive.

### 3.3. Optimization of Vertical Flow Assay Design and Experimental Parameters

**Figure 3:**
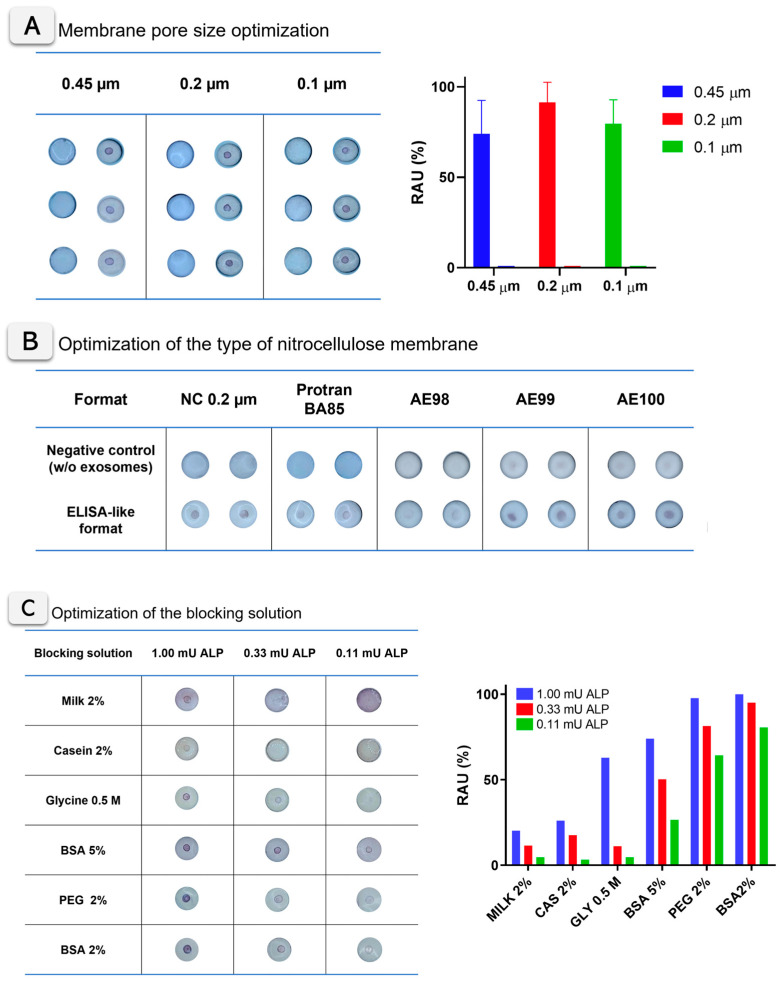
(**A**) Optimization of the pore size of nitrocellulose membrane. The images show the VFA cartridges of nitrocellulose membranes at different pore sizes (0.45, 0.2, and 0.1 µm), performed with 1 mU ALP. The bar plot shows the Image J colorimetric quantification of the visual signals, in relative area units, normalized by the maximum signal (n = 3). (**B**) Optimization of the type of nitrocellulose membrane. The images show the VFA cartridges with different nitrocellulose membranes for the ELISA-like format (n = 2). (**C**) Blocking solution optimization. Images of the VFA cartridges with ALP at 1 mU, 0.33 mU, and 0.11 mU of ALP. Different blocking agent solutions in tris buffer were tested (n = 1). The plot with the ImageJ colorimetric quantification of the visual signals, in relative area units (RAU), normalized by the maximum signal, is also shown. The negative controls are included in all cases.

The key experimental parameters of the VFA were optimized, including the selection of the nitrocellulose membrane type, pore size, and blocking solution. Three different pore sizes for the nitrocellulose membrane were tested, ranging from 0.1 to 0.45 µm, using ALP adsorbed on the membrane at an activity of 1 mU (500 mU mL^−1^). All experimental details are described in Section S5 (SI). Figure 3A shows the images of VFA cartridges obtained for three membrane pore sizes (0.45, 0.2, and 0.1 µm), with three replicates analyzed and quantified using ImageJ for each condition. No evident differences were observed by visual inspection between the cellulose nitrate membranes tested. A quantitative analysis and one-way ANOVA (*p* = 0.3904) confirmed that signal differences were not statistically significant, although the 0.2 µm membrane showed slightly higher mean intensity values. Importantly, no remarkable differences in flow rate, signal diffusion, or background development were detected across the tested membranes. As illustrated in Figure 3A, the 0.2 µm pore size was therefore selected as the optimal condition for subsequent experiments, providing the best balance between sensitivity, robustness, and reproducibility. The optimization of the type of membranes (including nitrocellulose, Protran BA85, and unbacked membranes AE98, AE99, and AE100) are described in Figure 3B. In this case, the experiment was based on the ELISA-like formats described in Figure 1B (based on antiCD81 mouse primary antibody and anti-mouse ALP-labeled secondary antibody), with exosomes derived from the SKBR3 cell line. In all cases, the corresponding negative control (processed without exosomes) was included. Figure 3B shows the images for the VFA cartridges, revealing clear differences between the membranes tested and the negative control in all cases. Particularly, unbacked membranes AE98, AE99, and AE100 showed less colored and more diffuse signals in all instances. On the other hand, Protran BA85 and nitrocellulose (NC) 0.2 membranes show clear visual positive areas, with evident differences between the negative (without exosomes) and positive samples. According to the results, in all cases, nitrocellulose (NC) membranes of a 0.2 µm pore size were selected in further experiments.

The blocking agent for the nitrocellulose membranes was also optimized using different buffers, as shown in Figure 3C. Casein and skimmed milk clearly reduce the appearance of the colored signal in the membrane. In the case of the glycine blocking solution, it provides clear purple signals at the test dot, although a yellowish background also appears. Regarding BSA and PEG blocking solutions, both provide optimal blocking of the background and high intensity purple signals. According to ImageJ quantification, the BSA 2% (*w*/*v*) solution was finally selected for further experiments. These optimization assays were conducted under parallel conditions to compare multiple parameters simultaneously, with the results presented as condition screening rather than statistical evaluation.

### 3.4. Vertical Flow Assay for Exosome Biomarker Quantification and Profiling

**Figure 4:**
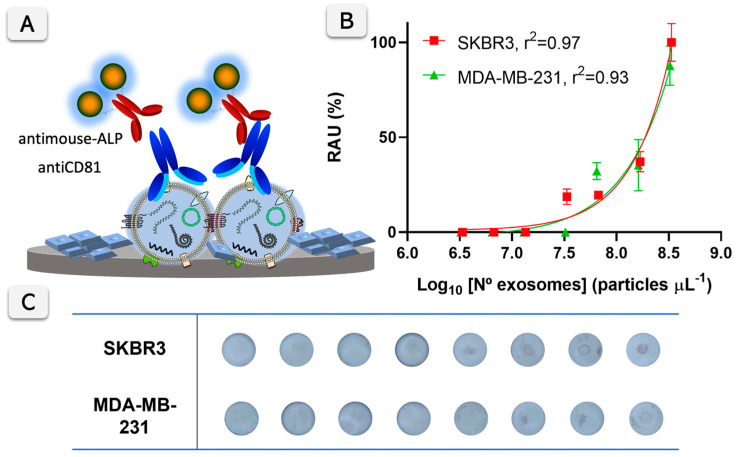
(**A**) illustrates the Vertical Flow Assay (VFA) quantification principle for exosome surface biomarker CD81. Exosomes bind to anti-CD81 antibodies, followed by detection using a secondary anti-mouse antibody conjugated with alkaline phosphatase (ALP), which produces an insoluble blue product upon substrate conversion. (**B**) presents calibration plots for exosomes derived from SKBR3 and MDA-MB-231 cells, with the relative absorbance units (RAU, %) plotted against the logarithm of the exosome concentration (particles µL^−1^). The fitted curves show good correlations (r^2^ = 0.97 for SKBR3 and r^2^ = 0.93 for MDA-MB-231). N = 3. (**C**) shows the corresponding membranes with the visual detection results for different exosome concentrations from SKBR3 and MDA-MB-231 cells, with increasing color intensity corresponding to higher exosome concentrations.

A calibration plot of the ELISA-like format was performed using an antiCD81 monoclonal antibody against the biomarker and a secondary ALP labeled antibody. The results are shown in Figure 4 for SKBR3 and MDA-MB-231-derived exosomes. The VFA signals are detectable at concentrations higher than 10^7^ exosomes µL^−1^ with the proposed protocol using the antiCD81 antibody. Specifically, the limit of detection estimated for the SKBR3-derived exosomes is 6.00 × 10^7^ particles µL^−1^, while for MDA-MB-231, it is 5.96 × 10^7^ particles µL^−1^. This LOD is calculated at the 15 min enzymatic reaction time. Accordingly, the reaction time of 15 min was selected as a compromise between assay sensitivity and total analysis time, ensuring efficient enzymatic signal development while maintaining a shorter assay duration compared to conventional formats. This reaction time represents a practical advantage of the proposed paper-based VFA, allowing rapid signal generation without compromising analytical performance. Nevertheless, the LOD could be further improved in situations where the exosome concentration is limited by extending the enzymatic reaction time, although this would come at the expense of a longer assay duration.

**Figure 5:**
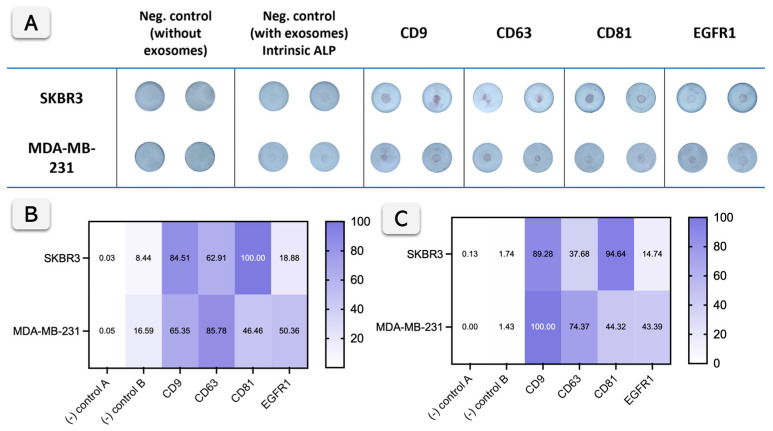
Exosome biomarker profiling using Vertical Flow Assay and comparison with bead-based flow cytometry. (**A**) shows the VFA results for exosome detection from SKBR3 and MDA-MB-231 cells using antibodies against CD9, CD63, CD81, and EGFR1. Negative controls include samples without exosomes (Control A) and samples with exosomes but no primary antibodies (Control B), with intrinsic alkaline phosphatase (ALP) activity shown in the second set of controls (n = 2). (**B**) presents a heat map quantifying the colorimetric signals from (**A**), indicating the relative intensity of biomarker detection for both cell lines. (**C**) shows the normalized results of bead-based flow cytometry for the same biomarkers, correlating with the VFA data and providing a comparative analysis of the detection sensitivity between the two methods.

Figure 5 shows the performance of the Vertical Flow Assay (VFA) for exosome profiling compared with bead-based flow cytometry. Raw data are shown in Figure 5A. Considering that exosomes can exhibit low intrinsic alkaline phosphatase (ALP) activity, as previously reported and quantified by our research group [1,2], it is mandatory to include a negative control with exosomes but without primary and secondary antibodies in the VFA. The specific ALP activity of exosomes was previously reported to be approximately 0.049 mU 10^9^ particles^−1^ for SKBR3 and 0.025 mU 10^9^ particles^−1^ for MDA-MB-231. However, this intrinsic activity becomes visually detectable only after about 30 min of enzymatic reaction and remains negligible under the conditions used in this study, as shown in Figure 5 (negative control B), where very low color development is observed. Compared with that background level, labeling with ALP-conjugated secondary antibodies markedly enhances the reaction rate, as higher amounts of enzymes are available to react with the substrate on the VFA surface, producing stronger signals.

The correlation between Figure 5B (VFA) and Figure 5C (flow cytometry, obtained by normalization of Figure S4, SI) demonstrates that both methods effectively detect exosome surface biomarkers (CD9, CD63, CD81, and EGFR1) from SKBR3 and MDA-MB-231 cells, with some variation in intensity. For CD9, CD63, and CD81, both methods show strong detection across the cell lines, with high intensities in both heat maps indicating a good correlation between the VFA and flow cytometry. For instance, the lower signal observed for CD63 in flow cytometry compared to the VFA can be attributed to methodological differences, mainly the use of distinct secondary antibodies and detection systems, which influence the efficiency of signal amplification. For EGFR1, both methods show weaker signals, suggesting it is less abundant, yet the correlation remains consistent between the two approaches.

EGFR1 positivity was relatively limited in the tested cell-line derived exosomes (13% in SKBR3; 37% in MDA-MB-231, Section 3.2, Figure S4), reflecting the heterogeneity of exosome cargo even among tumor-derived vesicle populations. These percentages correspond to the absolute values obtained from bead-based flow cytometry, while the data displayed in Figure 5C are shown in normalized form to facilitate comparison with the VFA results. This variability is consistent with the known heterogeneity of exosomes, as widely reported in the literature [31,32,33]. Furthermore, the lower EGFR1 signal observed in SKBR3 in both the VFA and flow cytometry is consistent with the reduced expression of this biomarker in SKBR3 cells and exosomes derived from SKBR3 cell lines, as previously reported [33]. In summary, while flow cytometry offers quantitative single-vesicle resolution and multiparametric fluorescence data, it requires complex instrumentation, dedicated software, and technical expertise. In contrast, the paper-based VFA provides a simple, accessible, and easy-to-interpret alternative that delivers rapid results without the need for specialized equipment. VFA thus provides a reliable, accessible, and cost-effective alternative for exosome biomarker detection.

The good correlation between the two methods suggests that VFA can be a valuable tool, particularly in settings where affordability and simplicity are priorities. This proof-of-concept was performed on purified exosomes to enable a controlled comparison with bead-based flow cytometry. Nonetheless, non-specific signals were mitigated by optimized blocking, membrane selection, and the use of appropriate negative controls, and the resulting profiles agreed with the flow cytometry data. Future work will evaluate matrix effects in crude supernatants and in clinical biofluids.

## 4. Discussion

The analysis of extracellular vesicles and particularly exosomes has gained increasing attention in the last decade, driven by their role in intercellular communication and their potential as non-invasive biomarkers in cancer and other diseases. However, their characterization remains technically demanding due to their nanometric size, heterogeneous composition, and low abundance in biological fluids. State-of-the-art approaches, including nanoparticle tracking analysis, electron microscopy, and flow cytometry, provide valuable information but require sophisticated instrumentation, long preparation times, and specialized expertise, which limit their widespread use [25]. The development of paper-based platforms aims to overcome these barriers by offering simple, rapid, and cost-effective alternatives that can extend exosome research beyond highly specialized laboratories.

This study demonstrated that a Vertical Flow Assay relying on an enzymatic colorimetric readout enables the reliable profiling of breast cancer-derived exosomes. By employing alkaline phosphatase conjugated secondary antibodies and NBT/BCIP as the substrate, the assay generated an insoluble precipitate on nitrocellulose membranes, which could be detected by the naked eye or quantified with a smartphone. This enzymatic method differs from the usual nanomaterial-based systems in LFA and VFA, which often depend on gold nanoparticles or fluorescent beads to improve sensitivity [5,6,13,14,15,16,17,18,20,21,22,23].

A key advantage of this system lies in its ability to generate biomarker expression profiles. The detection of tetraspanins CD9, CD63, and CD81, and the epithelial marker EGFR1 in exosomes from SKBR3 and MDA-MB-231 cell lines, showed good agreement with bead-based flow cytometry. Importantly, the relative expression patterns observed with the VFA paralleled those obtained with cytometry, supporting its validity as a semi-quantitative profiling tool. While flow cytometry remains the gold standard for detailed quantitative analysis, it requires the immobilization of exosomes onto magnetic particles to increase their detectable size, as well as benchtop flow cytometers and analysis software. In contrast, the VFA platform achieves comparable profiling with a simpler workflow and minimal infrastructure, highlighting its potential as a practical replacement for flow cytometry in contexts where accessibility and cost are critical considerations.

Among the few examples of VFAs applied to exosome analysis, one study reported the use of aptamer-functionalized gold nanostars combined with SERS detection for the multiplexed profiling of breast cancer exosomes [34]. This platform achieved high sensitivity but required complex nanomaterial synthesis and confocal Raman microscopy. In contrast, the enzymatic VFA presented here provides a simpler and more practical alternative, avoiding nanostructure fabrication and advanced instrumentation. It represents a cost-effective, reproducible, and portable solution that directly addresses the need for accessible exosome profiling in standard laboratory settings.

Although the present study was performed using exosomes derived from breast cancer cell lines, it should primarily be regarded as a proof-of-concept aimed at providing a simple alternative to flow cytometry for exosome profiling in cell culture research and characterization studies. In this context, the VFA could replace the need for bead-based flow cytometry when monitoring exosome production and biomarker expression in laboratory settings. Future studies will address clinical validation to assess the validity of exosome profiling in patient samples. The limit of detection obtained for the VFA (~6 × 10^7^ exosomes μL^−1^, 15 min reaction time) falls within the range of exosome concentrations reported in clinical biofluids such as plasma and serum (10^7^–10^11^ particles/μL), supporting its potential applicability for real biological samples [35,36,37].

In contrast to other approaches that rely on complex nanofabrication and advanced instrumentation—such as SERS–vertical flow hybrid biosensors [34], plasmonic metasensing platforms [38], or orthogonal SERS-FET devices for exosome detection [39]—this assay enables straightforward detection on a paper substrate and visual observation, which can be further quantified using a smartphone. This conceptual contribution lays the groundwork for subsequent clinical studies while highlighting the potential of paper-based VFAs as a low-cost and portable alternative. The inherent heterogeneity of exosome populations [31,32] further highlights the importance of including non-tumor controls and patient-derived samples in future studies to establish the clinical specificity of EGFR1 detection. The results also highlight the dual role of ALP in exosome analysis. Previous studies demonstrated intrinsic ALP activity in certain exosome populations [1], which can generate background signals if not properly controlled. By incorporating negative controls excluding antibodies, it was confirmed that the enzymatic signal in the VFA was dominated by the conjugated secondary antibody, thereby ensuring specificity. The choice of ALP also offers versatility, since its enzymatic activity can be coupled with a broad range of substrates producing stable, insoluble products suitable for paper-based immobilization [24]. Nonetheless, the relatively low turnover number of ALP compared to other enzymatic reporters represents a limitation in sensitivity, which could be addressed in future work by optimizing substrate chemistry or exploring multi-enzyme amplification strategies. VFAs expand the toolbox for exosome research and support future applications in cancer diagnostics and liquid biopsy.

## 5. Conclusions

This work presents the first paper-based Vertical Flow Assay for exosome analysis using alkaline phosphatase as an enzymatic reporter. The assay provides a straightforward, low-cost, and accessible strategy for exosome biomarker profiling, showing consistent results with conventional flow cytometry. By combining rapid visual detection with simple, smartphone-based quantification, this platform offers a practical alternative for laboratories seeking to study extracellular vesicles without relying on advanced equipment. While further improvements in sensitivity and validation with clinical samples are needed, future research should also expand the biomarker panel, integrate multiplexed detection, and explore broader applications. Overall, this work establishes the foundation for a cost-effective, scalable method for exosome profiling, capable of complementing or even replacing more complex techniques such as flow cytometry in routine laboratory practice.

## Data Availability

The data supporting the findings of this study are openly available in CORA.Repositori de Dades de Recerca.

## Supplementary Materials

The following supporting information can be downloaded at: https://www.mdpi.com/article/10.3390/bios15100694/s1; Figure S1: Schematic representation of the Vertical Flow Assay (VFA) cartridge components; Figure S2: EVs isolation protocol using differential ultracentrifugation; Figure S3: Characterization of purified EV samples from SKBR3 (A) and MDA-MB-231 (B) breast cancer cell lines by NTA and cryo-TEM; Figure S4. Dot blot (A) and histograms (B) of bead-based flow cytometry analysis of protein surface markers on exosomes derived from SKBR3 and MDA-MB-231; Table S1: Summary of VFA materials used in this work; Table S2: Particle concentration and protein concentration of exosome samples; Section S1: Experimental; Section S2: Cell culturing, exosome isolation and purification; Section S3: Characterization of exosomes by nanoparticle tracking analysis, cryogenic transmission electron microscopy and BCA protein assay; Section S4: Exosome biomarker profiling by bead-based flow cytometry; Section S5: Optimization of vertical flow assay design and experimental parameters; Section S6: Vertical Flow Assay for exosome biomarker quantification and profiling; Section S7. ImageJ colorimetric signal quantification.
