# Supplementary material for: Exosome Biomarker Profiling Using a Paper-Based Vertical Flow Assay
Source: Biosensors (Basel). 2025 Oct 14;15(10):694. doi: 10.3390/bios15100694 (PMC12563862; doi:10.3390/bios15100694)
Supplement: Supplementary file 1 [file biosensors-15-00694-s001.zip › biosensors-3874260-supplementary.pdf]

Supplementary information

Exosome Biomarker Profiling Using a Paper-Based Vertical Flow Assay

Arnau Pallarès-Rusiñol [1,2,‡], Jennifer Marfà [1,2,‡], Rosanna Rossi [1,2], Mercè Martí [2] and María Isabel Pividori [1,2,*]

[1] Grup de Sensors i Biosensors, Departament de Química, Universitat Autònoma de Barcelona, 08193, Bellaterra, Spain; arnau.pallares@uab.cat (A.P.-R.); jennifer.marfa@autonoma.cat (J.M.); rosanna.rossi@uab.cat (R.R.)
[2] Biosensing and Bioanalysis Group, Institute of Biotechnology and Biomedicine, Universitat Autònoma de Barcelona, Bellaterra, 08193, Spain; merce.marti@uab.cat (M.M.)
‡ These authors contributed equally to this work.
* Correspondence: isabel.pividori@uab.cat; Fax: +34 93 581 2379; Tel: +34 93 581 2806

S1. Experimental

Construction of vertical flow assay cartridges

The Vertical Flow Assay cartridges were constructed as shown in Figure S1 using the plastic cassettes obtained from Medmira. All details and references of all VFA materials tested are provided in Table S1. The plastic cassettes (panel 1 and 8) and plastic supports (panel 2, sized 28 × 20 × 1 mm, plus Ø 9 mm central hole) were obtained from Medmira. On the plastic support, a double-sided tape (panel 3) of the same dimensions was sticked. Then, the following membranes were sticked in order: i) a nitrocellulose membrane (panel 4, square or round pieces with Ø > 11 mm), a medium weight cotton linter pad (panel 5, square pieces sized 12 × 12 mm), plus a filter paper (panel 6, pieces sized 28 × 20 mm). Then, an absorbent pad (panel 7, pieces sized 28 × 20 mm) was putted below the membrane's unit. In the case of ELISA-like format described in Figure 1 (main manuscript), an additional non-absorbent separation membrane (square pieces sized 11 × 11 mm) between nitrocellulose (panel 4) and cotton linter pad (panel 5) was included. This fiber glass layer helped to reduce the background signals when using ALP-modified secondary antibodies.

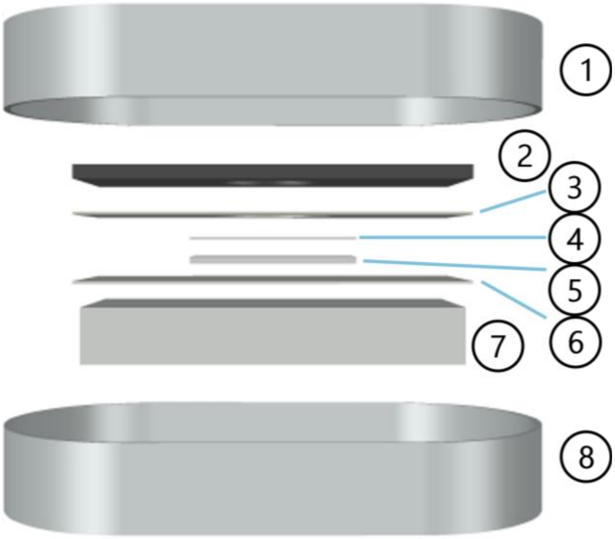

Figure S1. Schematic representation of the Vertical Flow Assay (VFA) cartridge components

Table S1. Summary of VFA materials used in this work

Material	Name	Supplier
**Cassettes and plastic support**	VFA cassettes	Ref. RVF VF-1-01, MedMira (Halifax, CA)
**Double-sided tape**	Double-sided tape	Ref. Medical Tape 1567, 3M (Saint Paul, MN, US)
**Nitrocellulose membrane**	NC – 0.2 µm pore size	Ref. 7182-001, Cytiva (München, GE)
	NC – 0.1 µm pore size	Ref. 7181-002, Cytiva
	NC – 0.45 µm pore size	Ref. 7184-001, Cytiva
	AE98	Ref. 10549916, Cytiva
	AE99	Ref. 10548081, Cytiva
	AE100	Ref. 10547017, Cytiva
	Protran BA85	Ref. 10402506, Cytiva
**Separation membrane**	VF2	Ref. 8145-2250, Cytiva
**Medium weight pad**	SP	Ref. CFSP203000, Merck-Millipore (Darmstadt, HE, GE)
	CF4	Ref. 8114-6621, Cytiva
**Filter paper**	Filter paper	(Local supplier) Density 64 g m^2
**Absorption thick pad**	CF7	Ref. 8117-2250, Cytiva

Buffers and solutions

All buffer and solutions were prepared with analytical reagent grade salts and ultrapure MilliQ water (Millipore® System, resistivity 18.2 MΩ·cm). Tris(hydroxymethyl)aminomethane (tris, ref. 252859), sodium phosphate dibasic (ref. 71636), potassium phosphate dibasic (ref. 795496), sodium chloride (ref. S3014), potassium chloride (ref. P3911), bovine serum albumin (ref. A4503), casein (ref. C7088), glycine (ref. 50046), and poly-ethylene glycol (ref. 8.07485) were purchased from Sigma-Aldrich (Merck KGaA, DE). Skimmed milk was purchased from local supplier (Nestlé Sveltesse).

The composition of the solutions was:

- Tris 1x buffer: 0.1 mol L^{-1} Tris-HCl, 0.15 mol L^{-1} NaCl, pH 7.4.
- PBS 1x buffer: 10 mmol L^{-1} Na_2HPO_4, 137 mmol L^{-1} NaCl, 2.7 mmol L^{-1} KCl, 1.8 mmol L^{-1} K_2HPO_4.
- PBS - 0.5% BSA buffer: 2% w/v BSA in PBS 1x buffer.

Specifically, the composition of blocking solutions tested for the VFA was:

- Skimmed milk 2%: 2% w/v of skimmed milk in Tris 1x buffer.
- Casein 2%: 2 % w/v of casein in Tris 1x buffer.
- Bovine serum albumin 2% and 5 %: 2% w/v, or 5% w/v, of BSA in Tris 1x buffer.
- Glycine 0.5M: 0.5 mol L^{-1} of glycine in Tris 1x buffer.
- Poly-ethylene glycol 2%: 2% w/v of PEG in Tris 1x buffer.

S2. Cell culturing, exosome isolation and purification

The cell lines used were breast cancer cell lines SKBR3 (ATCC, ref. HTB-30) and MDA-MB-231 (ATCC, ref. HTB-26). Expansion of cell population was carried out from 5 × 10^6 cells in T-175 flask containing 35 mL of Dulbecco's Modified Eagle's medium. The media were supplemented with 10% exosome-depleted fetal bovine serum (FBS) and 100 U mL^{-1} penicillin-streptomycin. The temperature was maintained at 37 °C in a humidified, concentrated CO_2 (5%) atmosphere. Once cells reached approximately 95% confluence on the T-175 flask, the culture supernatant was removed and stored at –20 °C until to exosome isolation.

Exosomes were purified according to what previously reported by our research group [1]. The supernatant from the SKBR3 and MDA-MB-231 cell lines were subjected to differential centrifugation as follows: 300 x *g* for 5 minutes (removal of residual cells), 2,000 x *g* for 15 minutes and 10,000 x *g* for 30 minutes (removal of cellular debris, large and medium-sized EVs). Then,

ultracentrifugation at 100,000 x *g* for 60 minutes was performed using a Beckman Coulter Optima L-80XP, either with a 70Ti or 50.2Ti rotor to pellet exosomes and other small EVs. After that, the supernatant was carefully removed, and crude exosome-containing pellets were resuspended in 1 mL of Tris 1x buffer (pH 7.4, 0.22 μm sterile-filtered) and pooled. The second round of the same ultracentrifugation setting was carried out, and the resulting exosome pellet resuspended in 500 μL (per each 100 mL of supernatant) of Tris 1x buffer (pH 7.4, 0.22 μm sterile-filtered) and stored at –20 °C. All centrifugation steps were performed at a temperature of 4 °C.

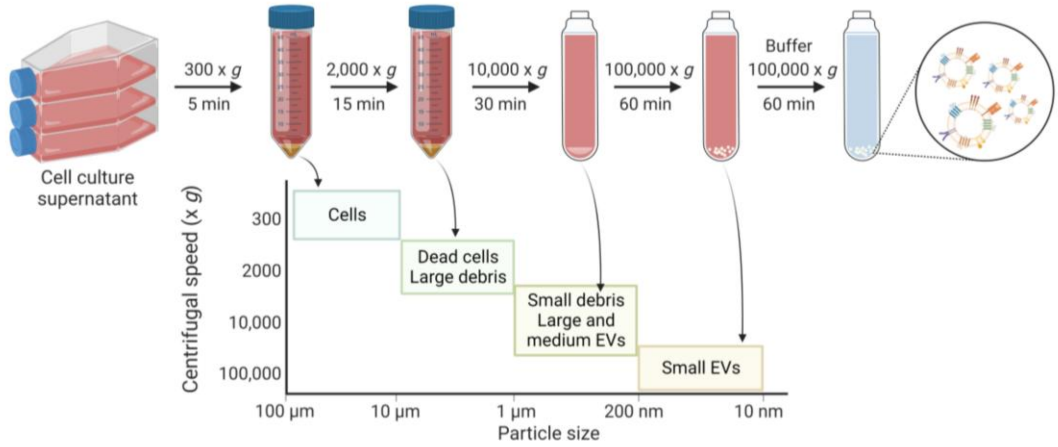

Figure S2. EVs isolation protocol using differential ultracentrifugation

S3. Characterization of exosomes by nanoparticle tracking analysis, cryogenic transmission electron microscopy and BCA protein assay

Nanoparticle tracking analysis (NTA) of exosomes samples from SKBR3 and MDA-MB-231 cell lines was used to analyze size distribution and particle concentration of the vesicles. Figure S3 shows similar size distribution in the three exosomes samples. On Table S2 shows particle concentration of the samples estimated by NTA, and total protein concentration estimated by BCA protein assay.

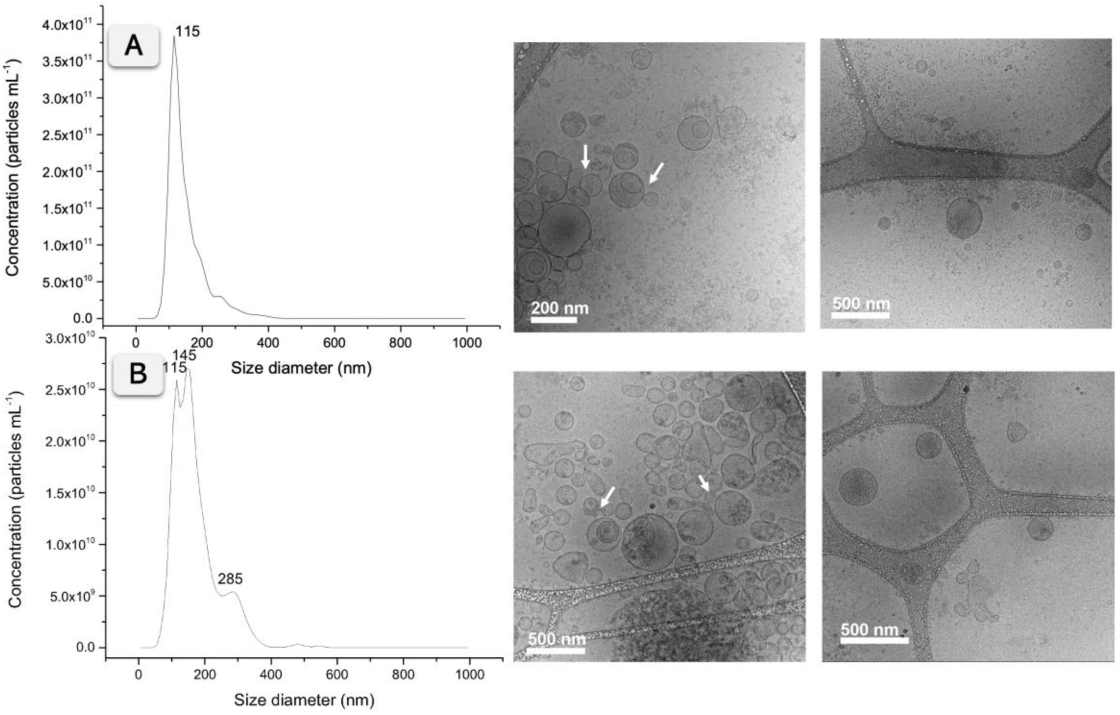

Figure S3. Characterization of purified EV samples from SKBR3 (A) and MDA-MB-231 (B) breast cancer cell lines by NTA and cryo-TEM

Table S2. Particle concentration and protein concentration of exosome samples

SAMPLE	PARTICLE CONCENTRATION (particles mL^{-1}) mean value /SD	PROTEIN CONCENTRATION (mg mL^{-1})
**SKBR3**	2.69 / 0.19 × 10^{12}	1.038
**MDA-MB-231**	3.24 / 0.04 × 10^{11}	0.324

S4. Exosome biomarker profiling by bead-based flow cytometry

Flow cytometry was used to estimate the presence of membrane protein markers of interest in the surface of exosomes derived from SKBR3 and MDA-MB-231 breast cancer cell lines. Specifically, the presence of tetraspanin receptors CD9, CD63 and CD81, and epithelial specific EGFR1 receptor were evaluated. The flow cytometry assay is based on the immobilization of exosomes on the surface of magnetic particles, to increase its size within the resolution of the flow cytometer.

The immobilization of exosomes on Dynabeads M450 tosylactivated superparamagnetic particles (MPs) were performed as follows: 3.5 × 10^{10} exosomes were added to 40 µL of MPs, equivalent to 1.6 × 10^{7} particles. The reaction was carried out in 0.1 mol L^{-1} borate buffer pH 8.5, in order to ensure the nucleophilic reaction by the amine group. The incubation step was

performed overnight with gentle shaking at 4 °C. After that, 0.5 mol L^{-1} glycine solution in PBS 1x buffer was added to ensure the blocking of the any remaining tosylactivated groups, by an incubation for 4 h at 25 °C. After that, the exosomes-modified magnetic particles (exosomes-MP) were resuspended in 160 μL of PBS 1x buffer to dilute the MPs suspension at 1 x 10^5 MPs per μL. The exosomes-MP were maintained at 4 °C until use.

The presence of the CD9, CD63, CD81 and EGFR1 biomarkers was investigated (Fig. S4). The indirect labelling of 5 × 10^5 exosome-modified MPs was performed by incubation of 100 μL (5 μg mL^{-1}) of the primary antibodies (*i.e.*, antiCD9, antiCD63, antiCD81, and antiEGFR1), for 60 min with gentle shaking at 4°C. After that, three washing steps with PBS buffer containing 0.5% BSA were performed. Afterwards, 100 μL (2 μg mL^{-1}) of antimouse-Cy5 secondary antibody were incubated for 30 min in the darkness with gentle shaking at 4°C. Again, three washing steps with PBS buffer containing 0.5% BSA were performed. The labelled MPs were resuspended in 1000 μL of PBS buffer.

Figure S4**A**, show dot blot plots of the bead-based flow cytometry measurements of SKBR3 and MDA-MB-231 exosomes-modified MPs. As insets in each plot, the positivity percentage of the sample is shown. In Figure S4**B**, the histograms representation of the positivity percentage is shown.

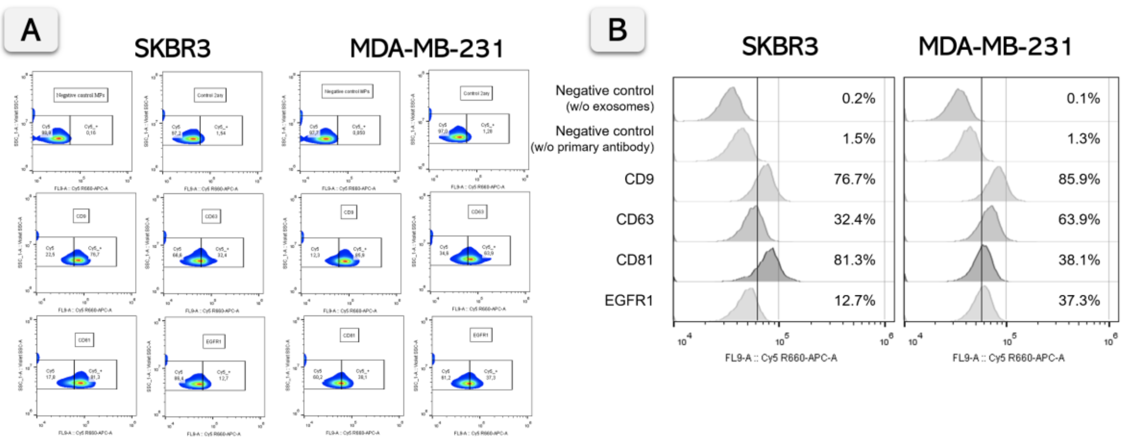

Figure S4. Dot blot (**A**) and histograms (**B**) of bead-based flow cytometry analysis of protein surface markers on exosomes derived from SKBR3 and MDA-MB-231

S5. Optimization of vertical flow assay design and experimental parameters

For the determination of ALP activity on samples immobilized on the membrane, standard VFA devices were constructed as detailed in S1. The following protocol was used for the experiments.

1. Immobilization: 2 μL of sample (typically ALP dilutions of 500, 166, and 55 mU mL^{-1}) was deposited at the center of the VFA nitrocellulose membrane, using a micropipette avoiding contact with the membrane surface. The VFA devices were air dried for 1 h at 4 °C.
2. Blocking: 200 μL of BSA 2% (w/v) in Tris buffer was added and incubated for 2h at 37°C in the oven.
3. Washing: 400 μL of Tris buffer.
4. Revealing: 10 μL of NBT/BCIP solution was added and incubated for different reaction times at 37°C.
5. Washing: 100 μL of Tris buffer.
6. Readout: Visual observation or imaging with a smartphone camera for further processing.

S6. Vertical Flow Assay for exosome biomarker quantification and profiling

For the ELISA-like determination of surface markers, ALP labelled antibodies were used as enzymatic reporters. It is important to note that in this case, a non-absorbent fibre glass membrane was added under the VFA nitrocellulose membrane, as detailed in S1. This additional layer helps to reduce the background signal coming from the unbounded ALP labelled antibodies that flow though the nitrocellulose. The following protocol was used for the experiments:

1. Immobilization: 2 μL of sample was deposited at the center of the VFA nitrocellulose membrane using a micropipette, avoiding contact with the membrane surface. The VFA were air dried for 1 h at 4 °C.
2. Blocking: 200 μL of BSA 2% (w/v) in Tris buffer was added and incubated for 2 h at 37 °C in the oven.
3. Incubation: a primary antibody (0.5 μg mL^{-1} in Tris buffer) and an ALP-labelled secondary antibody (30 ng mL^{-1} in Tris buffer) were incubated for 2 h at 37 °C. This enabled a one-step incubation of the antibodies in the VFA.
4. Washing: 200 μL of tris buffer.
5. Incubation: 100 μL of the antibody solution was added and incubated for 1h at 37°C.
6. Washing: 300 μL of Tris buffer.
7. Revealing: 10 μL of NBT/BCIP solution was added and incubated for 15 min at room temperature.
8. Washing: 100 μL of Tris buffer

9. Readout: Visual observation or imaging with a smartphone camera for further processing.

S7. ImageJ colorimetric signal quantification

The imaging of VFA cartridges was performed under controlled illumination to minimize interference during signal quantification. All cartridges corresponding to the same sample within an experiment were imaged simultaneously. Illumination was provided by a portable photographic light box equipped with white LED lights (1100 lm, color temperature 6000–6500 K). Images were captured at a fixed distance of 21 cm using a smartphone's rear camera (12-megapixel resolution), with autofocus enabled, 2× zoom, and the flashlight disabled during acquisition.

For colorimetric quantification, images were processed using ImageJ software (Fiji version). All images were converted to 8-bit format, and test spots were outlined with a circular selection tool. The area under each intensity peak was numerically integrated using the ImageJ Gel Analysis tool, and the resulting values were analyzed with GraphPad Prism v10.6.0 software.

References

(1) Moura, S. L.; Martín, C. G.; Martí, M.; Pividori, M. I. Multiplex Detection and Characterization of Breast Cancer Exosomes by Magneto-Actuated Immunoassay. *Talanta* **2020**, *211*, 120657. https://doi.org/https://doi.org/10.1016/j.talanta.2019.120657.
